# Conditional Process Analysis for Effective Lens Position According to Preoperative Axial Length

**DOI:** 10.3390/jcm11061469

**Published:** 2022-03-08

**Authors:** Young-Sik Yoo, Woong-Joo Whang

**Affiliations:** 1 Department of Ophthalmology, Uijeongbu St. Mary’s Hospital, College of Medicine, The Catholic University of Korea, Uijeongbu-si 11765, Korea; theblue07@naver.com; 2 Department of Ophthalmology, Yeouido St. Mary’s Hospital, College of Medicine, The Catholic University of Korea, Seoul 07345, Korea

**Keywords:** axial length, conditional process analysis, effective lens position, intraocular lens power calculation

## Abstract

Purpose: To predict the effective lens position (ELP) using conditional process analysis according to preoperative axial length. Setting: Yeouido St. Mary hospital. Design: A retrospective case series. Methods: This study included 621 eyes from 621 patients who underwent conventional cataract surgery at Yeouido St. Mary Hospital. Preoperative axial length (AL), mean corneal power (K), and anterior chamber depth (ACD) were measured by partial coherence interferometry. AL was used as an independent variable for the prediction of ELP, and 621 eyes were classified into four groups according to AL. Using conditional process analysis, we developed 24 structural equation models, with ACD and K acting as mediator, moderator or not included as variables, and investigated the model that best predicted ELP. Results: When AL was 23.0 mm or shorter, the predictability for ELP was highest when ACD and K acted as moderating variables (R2 = 0.217). When AL was between 23.0 mm and 24.5 mm or longer than 26.0 mm, the predictability was highest when K acted as a mediating variable and ACD acted as a moderating variable (R2 = 0.217 and R2 = 0.401). On the other hand, when AL ranged from 24.5 mm to 26.0 mm, the model with ACD as a mediating variable and K as a moderating variable was the most accurate (R2 = 0.220). Conclusions: The optimal structural equation model for ELP prediction in each group varied according to AL. Conditional process analysis can be an alternative to conventional multiple linear regression analysis in ELP prediction.

## 1. Introduction

The accuracy of intraocular lens (IOL) power calculation is a matter of great importance in cataract surgery [1,2]. IOL power is determined by three factors: preoperative biometric data (axial length (AL), anterior chamber depth (ACD), and mean corneal power (K)), the IOL power calculation formula, and the IOL constant [3]. Cataract surgeons have aimed to create an IOL formula for the determination of the ideal refractive outcome. The prediction of postoperative ACD or effective lens position (ELP) is the most important process in IOL power calculation, and IOL power calculation error is, for the most part, due to errors in predicting ELP [4].

Although more than 10 years have passed since the concept of the Haigis formula was introduced, it still shows high predictive accuracy [5,6]. The T2 formula, using only AL and K for ELP, shows the highest predictive accuracy [5,7]. However, there is an important limitation that the two formulas above are designed based on multiple linear regression analysis [8,9]. A multiple linear regression analysis, in principle, requires the independence of explanatory variables. However, ACD and K have significant relationships with AL, which can cause errors. Statistically, the explanatory variables used in ELP prediction are considered to have a collinearity problem [10,11].

Hayes presented dozens of models in PROCESS macros for conditional process analysis [12,13,14]. Conditional process analysis includes not only independent variables, but also the concept of a mediating variable and a moderating variable. Using this method, we can solve the problem of multicollinearity and identify relationships between explanatory variables and develop a more accurate structural equation model for a dependent variable.

In this study, considering that the formula yielding excellent accuracy differs according to AL, we divided a total of 621 eyes into four groups according to AL. We determined the ideal model for predicting ELP in each group on the basis of conditional process analysis and the results were compared with existing IOL formula derived from a multiple linear regression analysis.

## 2. Materials and Methods

This retrospective case series study included 621 eyes of 621 patients who underwent uneventful and micro-coaxial phacoemulsification cataract surgery without any intraoperative complications between March 2018 and September 2019. None of the patients had a history of ocular disease, previous ocular surgery, or general disorders affecting the cornea. Exclusion criteria were amblyopia, corneal opacity, glaucoma, retinal disease, history of ocular inflammation, history of ocular trauma, and history of exposure to other intraocular surgeries. The study methods adhered to the tenets of the Declaration of Helsinki for use of human participants in biomedical research. The Institutional Review Board (IRB #SC20RASI0071) for Human Studies at Yeouido St. Mary’s Hospital approved this study, and informed consent was exempted by IRB of Yeouido St. Mary’s Hospital.

Preoperative biometric measurements, such as K of anterior surface, ACD, and AL, were obtained with an IOLMaster optical biometer (version 5, Carl Zeiss, Oberkochen, Germany) to calculate IOL power. All procedures were performed by two surgeons (H.S. Kim and W.J. Whang). All patients underwent cataract surgery through a 2.2 mm micro coaxial incision under topical anesthesia (proparacaine hydrochloride 0.5%, Alcaine, Alcon). After performing continuous curvilinear capsulorhexis with an intended diameter of 5.0 mm and hydrodissection, phacoemulsification of the nucleus was performed using an OZil torsional handpiece with the Centurion vision system (Alcon, Fort Worth, TX, USA). Following phacoemulsification, the intraocular lens (ZCB00, Johnson & Johnson Vision, Santa Ana, CA, USA) was inserted into the capsular bag using an injector and disposable cartridge system before removing the ophthalmic viscosurgical device. Finally, a balanced salt solution was injected into the corneal incision site with stromal hydration. After the surgery, postoperative antibiotic and corticosteroid eye drops were used four times daily and tapered over a month.

Subjective refraction was measured 3 months postoperatively with manifest refraction by an experienced ophthalmologist (J. Y. Lee) and ELP was back-calculated using the following thin-lens formula [15]:

$$\mathrm{IOL}\;\mathrm{power}=\frac{1336}{\mathrm{AL}-\mathrm{ELP}}-\frac{1336}{\frac{1336}{Z}-\mathrm{ELP}}$$

$$Z=\frac{\left(nc-1\right)\times 1000}{r}+\frac{1000}{\frac{1000}{PostRx}-VD}$$

where *nc* is the fictious corneal refractive index (1.3315), *r* (millimeter) is the mean value of the preoperative corneal radius, *PostRx* is the postoperative spherical equivalent, and *VD* (millimeter) is the vertex distance.

The 621 eyes were stratified into 4 subgroups to investigate the appropriate structural equation model according to the preoperative AL:

1. AL ≤ 23.0 mm (*n* = 144)
2. 23.0 mm < AL ≤ 24.5 mm (*n* = 291)
3. 24.5 mm < AL ≤ 26.0 mm (*n* = 119)
4. AL > 26.0 mm (*n* = 67)

The ELP prediction error was defined as the value calculated by subtracting the predicted ELP from the back calculated ELP based on the thin-lens formula described above. Conditional process analysis was defined as the method for calculating ELP prediction in the present study. The accuracy of refractive outcomes (prediction error (PE), median absolute error (MedAE), and mean absolute error (MAE)) using conditional process analysis was compared to those using the Haigis formula. Refractive outcomes using the Haigis formula were calculated using an optimized IOL constant for the IOLMaster (ZCB00; a0 = −1.302, a1 = 0.210 and a2 = 0.251 based on ULIB site) and the zeroing of ME was performed based on the analysis methods suggested by Hoffer et al. [16]. PE was defined as the actual postoperative spherical equivalent minus the predicted spherical equivalent using the IOL power actually implanted. MedAE and MAE were the median and the average from the absolute value of the PE, respectively. The percentages of eyes with PE within ±0.25 D, ±0.50 D and ±1.00 D were also obtained.

### Statistical Analysis

Pearson’s correlation tests were performed to determine the strength of association between AL and other variables. A multiple linear regression test was used to develop an ELP prediction equation using AL and ACD. A PROCESS macro for SPSS statistical software (version 21.0, SPSS, Inc., Chicago, IL, USA) was used for conditional process analysis. In model templates for the PROCESS macro, we chose models that consist of two or three explanatory variables. Additionally, under the assumption that the AL is the most important variable for ELP prediction, AL was set as an independent variable and ELP was set as a dependent variable. ACD and K were used as mediating variables or moderating variables, or not used. The models adopted in this study are listed in Table 1. We found the ideal combination with the highest R2 value in 24 cases derived from 12 models in each subgroup.

## 3. Results

Demographic data for a total of 621 eyes are listed in Table 2. AL ranged from 21.41 to 30.60 mm, with a mean of 24.08 ± 1.54 mm; ACD ranged from 2.02 to 4.29 mm, with a mean of 3.20 ± 0.41 mm; and K ranged from 40.30 to 49.28 diopter, with a mean of 44.12 ± 1.42 diopter. ELP ranged from 3.67 to 8.76 mm, with a mean of 5.16 ± 0.63 mm. The three preoperative parameters and ELP in the four subgroups are listed in Table 3.

**Figure 1:**
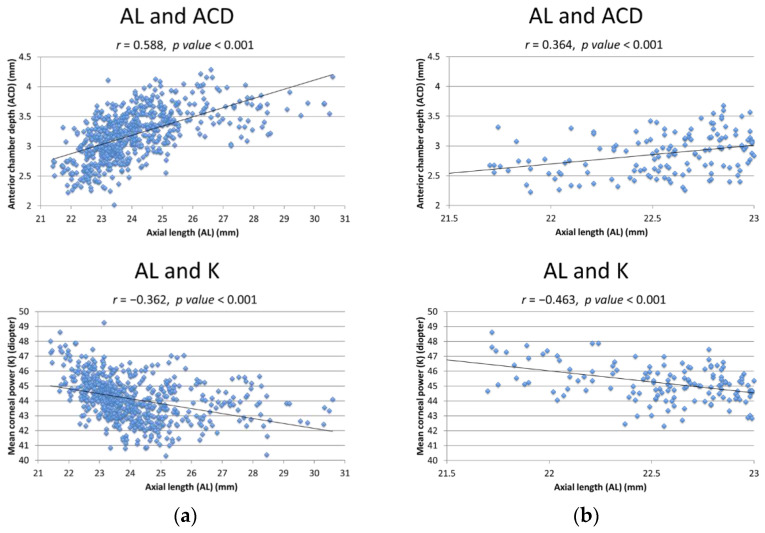

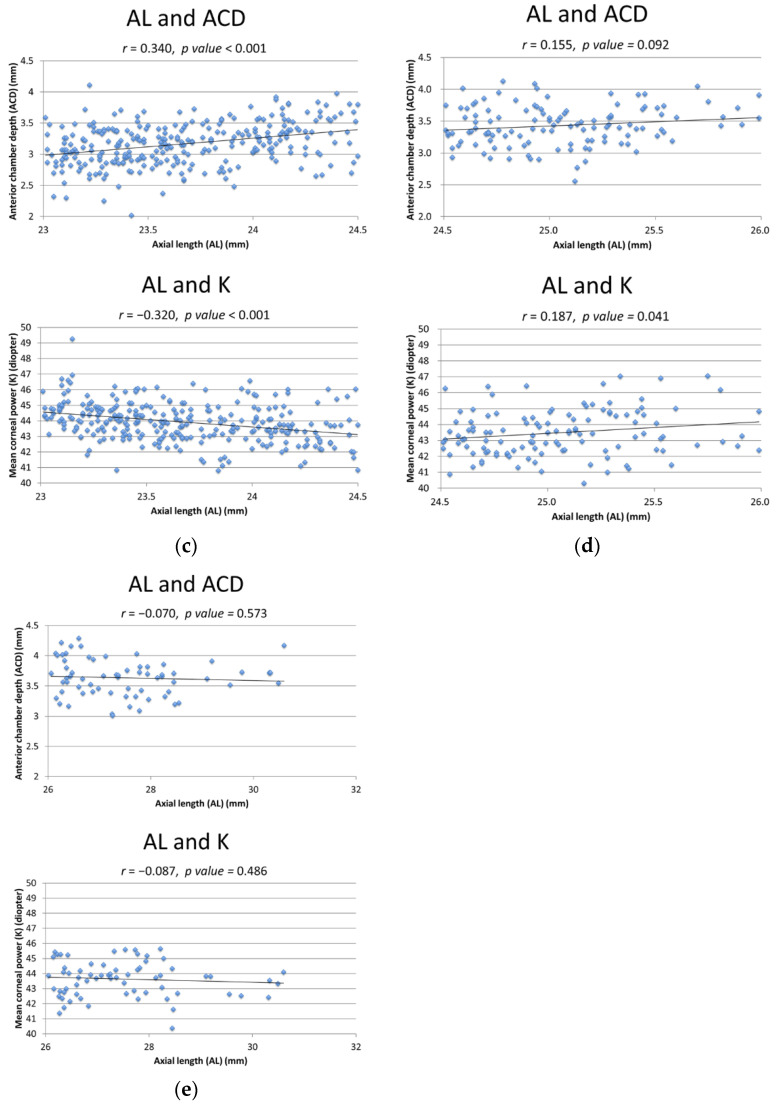
The relationships between axial length (AL) and other variables for structural equation models. (**a**) Total 621 eyes; (**b**) AL ≤ 23.0 mm; (**c**) 23.0 mm < AL ≤ 24.5 mm; (**d**) 24.5 mm < AL ≤ 26.0 mm; (**e**) AL > 26.0 mm. K = mean corneal dioptric power; ACD = anterior chamber depth.

Figure 1 shows the relationship between AL and the other two variables used in structural equation models. When all the 621 eyes were analyzed at once, AL and ACD showed a positive correlation, and AL and K showed a negative correlation (r = 0.588; *p* < 0.001 and −0.362; *p* < 0.001, respectively). However, in the subgroup analysis, both parameters showed significant correlations when AL was 24.5 mm or shorter (all *p* < 0.001).

**Figure 2:**
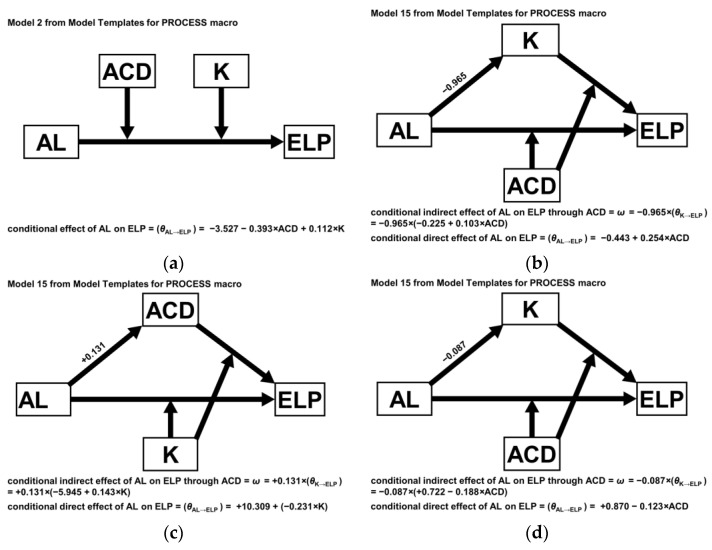
The structural equation models for the prediction of effective lens position (ELP) in each range of axial length (AL). (**a**) AL ≤ 23.0 mm; (**b**) 23.0 mm < AL ≤ 24.5 mm; (**c**) 24.5 mm < AL ≤ 26.0 mm; (**d**) AL > 26.0 mm. K = mean corneal dioptric power; ACD = anterior chamber depth.

Figure 2 demonstrates structural equation models with the highest R^2^ value among 24 cases in four subgroups. When AL was shorter than 23.0 mm, the model where both K and ACD acted as the moderating factor (Model 2 from PROCESS macro) showed the highest R^2^ value (0.217, *p* < 0.001, Figure 2a). When AL ranged from 23.0 to 24.5 mm, the R^2^ value (0.217, *p* < 0.001) was highest with Model 15 (K as a mediating variable, ACD as a moderating variable in both the process from AL to ELP and the process from K to ELP, Figure 2b). In the range of AL between 24.5 mm and 26.0 mm, unlike the above, when ACD acts as a mediating variable and K acts as a moderating variable in the processes of influencing ELP, the R^2^ value (0.220, *p* < 0.001) is highest (Figure 2c). Figure 2d shows the model when AL was longer than 26.0 mm. The predictability is highest (R^2^ value = 0.401, *p* < 0.001) with K as a mediating variable and ACD as a moderating variable.

Table 4 shows regression formulas derived from a multiple linear regression analysis using AL and ACD in a total of 621 eyes and conditional process analysis.

The mean ELP prediction error and the predictive accuracy from the above two analysis methods are listed in Table 5. The results from conditional process analysis yielded lower standard deviation (SD) of mean ELP prediction error, lower SD of mean prediction error, lower median absolute error and lower mean absolute error compared with results from a multiple regression test. It also produced higher percentages within ±0.25, ±0.50, and ±1.00 diopter.

## 4. Discussion

The results of this study demonstrated that the optimal structural equation models, consisting of preoperative parameters for the prediction of ELP, were different according to preoperative AL. The regression equations derived from the conditional process analysis could be developed into an IOL calculation formula with high predictive accuracy.

Recently, the Barret Universal II formula, the EVO (Emmetropia Verifying Optical) formula, the Hill-RBF (radial basis function) formula, and the Kane formula have been introduced, and the accuracy of these new formulas has been reported to be improved compared to existing ones [17]. Unfortunately, the detailed mechanism of these new formulas is not known. In particular, both the Hill-RBF and the Kane formula are well known for using artificial intelligence algorithms. In addition, the possibility of IOL calculation formulas using multilayer perceptron, which is another form of artificial intelligence, has been suggested [18]. Even if the design mechanism of artificial intelligence is clearly disclosed, artificial intelligence algorithms usually have multiple hidden layers, so surgeons cannot understand the detailed calculation process [19]. This effect is called the “black box effect” and has been pointed out as a disadvantage in equations through artificial intelligence. Of course, the accuracy of IOL power calculation through artificial intelligence is already high, and there is no doubt that it will develop further in the future. However, through the results of this study, we would emphasize that the accuracy of the formula can be improved through conditional process analysis, and that the information on the detailed calculation process can be clearly provided to anyone.

The formula that produces high accuracy for postoperative refractive outcomes differs according to preoperative AL. When the AL is markedly short or long, the accuracy of the IOL calculation formula is lower than that in eyes with AL in the normal range. The Hoffer Q formula was more accurate than the other formulas in cases of eyes with short AL (AL < 22.0 mm) [20,21]. Wang et al. advocated the use of the Haigis formula for the determination of IOL power in myopia with long AL [22], so we divided a total of 621 eyes into four subgroups in 1.5 mm increments according to AL.

The correlation between postoperative refraction error and AL and K has also been studied in patients that underwent cataract surgery after refractive surgery. Recently, an advanced lens measurement approach (ALMA) was proposed to improve the accuracy of postoperative refraction error by Rosa et al. [23]. They showed the improvement of R Factor [24] and ALxK methods [25] by applying ALMA, which is a mixed theoretical regression method based on the SRK-T formula.

Almost all theoretical formulas for IOL power calculation are based on the use of a simplified eye model with a thin cornea and an IOL model. [26]. With this approach, the power of the IOL can be easily calculated using the Gauss equation in paraxial optics. [27]. ELP is back-calculated by “predicting” the effective ACD value with the actual postoperative refraction of a given data set. Therefore, ELP is formula-dependent and does not need to consider the real postoperative IOL position in terms of the eye’s anatomy [28]. Models based on statistically analyzed relationships between some or all of the previously mentioned preoperative measurements of the eye and postoperative IOL position have been used to predict ELP in preoperative settings. In 1975, Fyodorov et al. [29] derived an equation based on the individual eye’s keratometry and AL to estimate ELP. Third-generation formulas, including the Hoffer Q, [27] Holladay 1, [28] and SRK/T formulas, [30] use AL and K to predict ELP and IOL power calculation, and the main difference among these formulas is the predicted value of ELP. As ACD values can be measured accurately after the development of slit-scan technology, a fourth-generation formula, the Haigis formula, was developed to estimate ELP with the AL and ACD values, [31,32]. The commonality of the various formulas used from the past to the present is that the AL is considered to be the most important factor in ELP prediction. Therefore, in this study, the AL was set as a constant independent variable in the ELP prediction process.

Sheard et al. concluded that the SRK/T formula has non-physiologic behavior that contributes to IOL power prediction errors [9]. Specifically, Reitblat et al. found that the SRK/T formula induced myopic results in eyes with a mean K greater than 46.0 diopter, and hyperopic results in eyes with a mean K lower than 42.0 diopter [33]. In contrast, the Haigis formula, which does not consider corneal steepness during ELP calculation, causes myopic outcomes in flat corneas. This tendency has also been proven in large-scale research by Melles et al. [6]. However, previous studies have concluded that there is no significant association between mean K and postoperative IOL position [11,24]. In this study, we attempted to investigate the effects of K on ELP and to determine why the conclusions of the above-mentioned studies are controversial. We found a highly predictable model by setting K as variables that mediate or moderate the action of AL.

In general, ACD is positively correlated with AL, and this was reconfirmed in this study. In other words, ACD and AL inevitably have the problem of multicollinearity. However, the results of this study documented the correlation between the two parameters varies depending on the range of AL. ACD, like K, also acted as a mediator or a moderator depending on the AL and has been found to be an essential element in ELP prediction.

There are some limitations in this study. We did not evaluate other factors such as lens thickness (LT) or corneal diameter. Recently, there has been a growing interest in the thickness of the crystalline lens and LT is considered in newly developed IOL power calculation formulas. Norrby et al. concluded that LT was not an essential factor and ACD alone would predict the postoperative IOL position accurately [11]. In the above study, they used a partial least squares (PLS) regression test and LT, as an independent variable, may not be as effective. However, it could act as a factor that mediates or modulates the effect of ACD, and it is also expected that this will further improve the accuracy of the equation. Corneal diameter was significantly correlated with postoperative IOL position in another study [34]. Therefore, it is thought that corneal diameter can act as a mediator in the process from AL to ELP by itself (direct effect), or can act as a factor that mediates or moderates the effect of K (indirect effect). Corneal asphericity is another candidate. The prediction error from modern IOL calculation formulas was influenced by corneal asphericity [35,36]. Corneal asphericity could be a mediating or moderating variable in the process where K or corneal diameter affects ELP. Various models have already been introduced that can handle many variables using conditional process analysis. If new variables are included in conditional process analysis, the predictive accuracy of the equation would be further improved. A second limitation is the relatively small population. The main problem that can arise from the small population is the overfitting of the derived equation. This “overfitting” problem would be solved by increasing the number of the study populations in future studies. In addition, ideal models were found by classifying four groups in 1.5 mm increments in this study. If the number of populations is sufficient, we can find more optimized models by reducing the units of AL and increasing the number of subgroups. Lastly, the analysis of refractive outcomes based on postoperative refraction could be affected by the bias in the preoperative measurement of AL, as shown by the decrease in AL measured using an IOLMaster after cataract surgery reported by De Bernardo M et al. [37].

In conclusion, depending on the preoperative AL, the ideal structural equation model for ELP prediction derived from conditional process analysis differs. Conditional process analysis can be an alternative to conventional multiple linear regression analysis in ELP prediction and IOL power calculation.

### 4.1. What Was Known

- The formula that produces high accuracy for postoperative refractive outcomes differs according to preoperative axial length.
- The prediction of effective lens position is the most important process in modern IOL calculation formulas.

### 4.2. What This Parer Adds

- In conditional process analysis, the ideal model for the prediction of effective lens position varies according to preoperative axial length.
- Structural equation modeling from conditional process analysis is an effective tool for the prediction of an effective lens position.

## Figures and Tables

**Table 1:** Models with 3 variables for the prediction of effective lens position (ELP). The axial length (AL) was set as an independent variable and ELP was set as a dependent variable. Each model was provided by PROCESS macro for conditional process analysis [12,13,14].

Model Number from PROCESS Macro [12,13,14]	Case	Mediating Variable	Moderating Variable
1	case 1		ACD
	case 2		K
2	case 1		ACD and K
3	case 1		ACD as a primary variable K as a secondary variable
	case 2		K as a primary variable ACD as a secondary variable
4	case 1	ACD	
	case 2	K	
	case 3	ACD and K	
5	case 1	ACD	K
	case 2	K	ACD
6	case 1	ACD as a first variable K as a second variable	
	case 2	K as a first variable ACD as a second variable	
7	case 1	ACD	K as a moderating variable for ACD
	case 2	K	ACD as a moderating variable for K
8	case 1	ACD	K as a moderating variable for ACD and ELP
	case 2	K	ACD as a moderating variable for K and ELP
14	case 1	ACD	K as a moderating variable in the process from ACD to ELP
	case 2	K	ACD as a moderating variable in the process from K to ELP
15	case 1	ACD	K as a moderating variable in the processes from ACD to ELP and from AL to ELP
	case 2	K	ACD as a moderating variable in the processes from K to ELP and from AL to ELP
58	case 1	ACD	K as a moderating variable in the processes from AL to ACD and from ACD to ELP
	case 2	K	ACD as a moderating variable in the process from AL to K and from K to ELP
59	case 1	ACD	K as a moderating variable in the processes from AL to ACD, from ACD to ELP, and from AL to ELP
	case 2	K	ACD as a moderating variable in the process from AL to K, from K to ELP, and from AL to ELP

ACD = anterior chamber depth; K = mean corneal dioptric power.

**Table 2:** Demographic data in this study.

	Number	Mean	Min.	Max.
Axial length (mm)	621	24.08 ± 1.54	21.41	30.60
Anterior chamber depth (mm)	621	3.20 ± 0.41	2.02	4.29
Mean keratometry (diopter)	621	44.12 ± 1.42	40.30	49.28
Age	621	69.46 ± 10.20	37	98
Effective lens position (mm)	621	5.16 ± 0.63	3.67	8.76
IOL power (diopter)	621	19.98 ± 3.47	5.5	27.0
Postoperative spherical equivalent of refraction (diopter)	621	−0.85 ± 1.06	−4.13	1.00

**Table 3:** Demographic data in 4 subgroups classified according to preoperative axial length (AL).

		Number	Mean	Min.	Max.
AL ≤ 23.0 mm	AL (mm)	144	22.42 ± 0.39	21.41	23.00
	ACD (mm)	144	2.86 ± 0.34	2.23	3.68
	K (D)	144	45.26 ± 1.24	42.32	48.63
	ELP (mm)	144	4.75 ± 0.40	3.67	5.69
23.0 mm < AL ≤ 24.5 mm	AL (mm)	291	23.67 ± 0.41	23.01	24.50
	ACD (mm)	291	3.17 ± 0.33	2.02	4.11
	K (D)	291	43.93 ± 1.23	40.82	49.28
	ELP (mm)	291	5.02 ± 0.39	3.95	6.56
24.5 mm < AL ≤ 26.0 mm	AL (mm)	119	25.05 ± 0.37	24.51	25.99
	ACD (mm)	119	3.43 ± 0.31	2.56	4.13
	K (D)	119	43.48 ± 1.44	40.30	47.05
	ELP (mm)	119	5.38 ± 0.48	4.21	7.05
AL > 26.0 mm	AL (mm)	67	27.50 ± 1.17	26.06	30.60
	ACD (mm)	67	3.64 ± 0.30	3.01	4.29
	K (D)	67	43.64 ± 1.17	40.38	45.65
	ELP (mm)	67	6.25 ± 0.76	4.98	8.76

ACD = anterior chamber depth; K = mean corneal dioptric power; ELP = effective lens position; D = diopter.

**Table 4:** Regression formulas for prediction of effective lens position according to preoperative axial length.

	Regression Formula for ELP Prediction	
	Haigis Formula	Conditional Process Analysis
AL ≤ 23.0 mm	−2.123 + 0.288 × AL + 0.107 × ACD	78.662 − 3.527 × AL + 8.784 × ACD − 2.399 × K −0.393 × AL × ACD + 0.112 × AL × K
23.0 mm < AL ≤ 24.5 mm		25.237 − 0.443 × AL − 10.495× ACD − 0.225 × K + 0.254 × AL × ACD + 0.103 × ACD × K
24.5 mm < AL ≤ 26.0 mm		−236.636 + 10.309 × AL − 5.945 × ACD + 5.380 × K −0.231 × AL × K + 0.143 × ACD × K
AL > 26.0 mm		−49.768 + 0.870 × AL + 11.757 × ACD + 0.722 × K −0.123 × AL × ACD − 0.188 × ACD × K

ACD = anterior chamber depth; AL = axial length; K = mean corneal dioptric power; ELP = effective lens position.

**Table 5:** Predictive outcomes derived from the Haigis formula and conditional process analysis.

		Haigis Formula	Conditional Process Analysis
Mean ELP prediction error (D)		0.000 ± 0.424	0.000 ± 0.396
Mean prediction error (D)		0.000 ± 0.521	0.000 ± 0.488
Median absolute error (D)		0.344	0.331
Mean absolute error (D)		0.408 ± 0.324	0.386 ± 0.299
Percentages of Eyes within (D)	±0.25	39.1	39.8
	±0.50	68.6	70.9
	±1.00	94.0	95.3

ELP = effective lens position; D = diopter.

## Data Availability

Data collected for this study, including individual patient data, will not be made available.
